# Factors associated with utilization of antenatal care services in Balochistan province of Pakistan: An analysis of the Multiple Indicator Cluster Survey (MICS) 2010

**DOI:** 10.12669/pjms.316.8181

**Published:** 2015

**Authors:** Abdul Ghaffar, Sathirakorn Pongponich, Najma Ghaffar, Tahir Mehmood

**Affiliations:** 1 Dr. Abdul Ghaffar, MBBS, Ph.D. College of Public Health Sciences, Chulalongkorn University, Bangkok, Thailand. Planning Officer, Health Department, Government of Balochistan, Pakistan; 2 Dr. Sathirakorn Pongponich Ph.D. College of Public Health Sciences, Chulalongkorn University, Bangkok, Thailand; 3 Dr. Najma Ghaffar, FCPS. Department of Gynaecology and Obstetrics, Bolan Medical Complex Hospital, Quetta, Pakistan; 4 Dr. Tahir Mehmood, Ph.D. Statistics, Basic Science, Riphah International University, Islamabad, Pakistan. Biostatistics, Dept. of Chemistry, Biotechnology & Food Sciences, Norwegian University of Life Sciences, Norway

**Keywords:** Antenatal care services, Associated factors, Multiple Indicator Cluster Survey analysis, Utilization

## Abstract

**Objective:**

The study was conducted to identify factors affecting the utilization of Antenatal Care (ANC) in Balochistan Province, Pakistan.

**Methods:**

Data on ANC utilization, together with social and economic determinants, were derived from a Multiple Indicator Cluster Survey (MICS) conducted in Balochistan in 2010. The analysis was conducted including 2339 women who gave birth in last two years preceding the survey. The researchers established a model to identify influential factors contributing to the utilization of ANC by logistic regression; model selection was by Akaike Information Criterion (AIC) and Bayesian Information Criterion (BIC).

**Results:**

Household wealth, education, health condition, age at first marriage, number of children and spouse violence justification were found to be significantly associated with ANC coverage. Literate mothers are 2.45 times more likely to have ANC, and women whose newborns showed symptoms of illness at birth that needed hospitalization are 0.47 times less likely to access ANC. Women with an increase in the number of surviving children are 1.07 times less likely to have ANC, and those who think their spouse violence is socially justified are 1.36 times less likely to have ANC. The results draw attention towards evidence based planning of factors associated with utilization of ANC in the Balochistan province.

**Conclusion:**

The study reveals that women from high wealth index and having education had more chances to get ANC. Factors like younger age of the women at first marriage, increased number of children, symptoms of any illness to neonates at birth that need hospitalization and women who justify spouse violence had less chances to get ANC. Among components of ANC urine sampling and having tetanus toxoid (TT) in the last pregnancy increased the frequency of visits. ANC from a doctor decreased the number of visits. There is dire need to reduce disparities for wealth index, education and urban/rural living.

## INTRODUCTION

Pakistan is facing challenges to reduce the high burden of maternal, and under 5 year’s child mortality.^1^ Globally, the maternal mortality ratio (MMR) has been reduced by 47% over the last twenty years, from 400 maternal deaths per 100,000 live births in 1990 to 210 in 2010. Currently, MMR in developing countries is 14 times higher than in the developed countries^2^ and ANC coverage (at least one visit with a doctor, nurse or midwife during pregnancy) have increased from 63% to 81% between 1990 and 2011. Some of the struggling countries, like many in southern Asia, northern Africa and western Asia have improved in the last ten years, and regions such as the Caribbean, eastern Asia, Latin America and south eastern Asia have now reached ANC coverage rates of 90% or more.^3^

Several factors influences utilization of ANC including wealth index, education, living in urban or rural areas and quality of services provided and delivering timely and efficient health care choices to the women of reproductive age.^4^ Fertility rate, number of children and family size also influence ANC utilization.^4^ Health services provision and planning in Balochistan Province are always challenging for health managers due to its difficult and harsh topography and inaccessible and scattered population.^5^

MDG data from Balochistan pinpoints a number of extremely worrying outcomes: a persistently high total fertility rate; low contraceptive prevalence across province outside of the capital city Quetta; a decline in the percentage of pregnant mothers using antenatal care services; and slow progress in increasing the presence of skilled birth attendants.^3^ Due to these factors the maternal mortality ratio is high in the province and appears to have increased between 2004 and 2007.^4^

Recent and reliable data is also not available to know whether these negative trends have been arrested or continue their contribution to an extremely dire situation for pregnant mothers, particularly those in rural Baluchistan. According to the MICS Balochistan 2010 39% of mothers received ANC from a skilled health provider at least once, and 11% at least four times from any provider during their last pregnancy in the two years preceding the survey.^6^-^8^

After the 18^th^ amendment of the constitution of Pakistan, the role of provinces in providing a healthy community in the country has increased. Provinces have to develop short and long-term strategies to address their health issues. The Balochistan MICS 2010 was undertaken to evaluate the progress of MDG 4 and 5 and of women and children health, and ultimately to provide assistance to the government of Balochistan for creating long-term strategic plans. Despite many projects and programs from the federal government, international donors, non-governmental organizations, the health system of Balochistan province is unable to provide standard primary health care services and is unable to achieve any of the MDGs by 2015.^4^

Balochistan Province is the largest by land and is the least developed province in Pakistan^8^. In this area the urbanization rate has been slow compared to other regions during last six decadesand majority of the population is uneducated and still living in their traditional tribal system.^8^ Their conservative values have great cultural influence on the community even in this modern era. They have occupations like animal husbandry, agriculture and government jobs for the males. Baloch men are dominant in every aspect of life. Women and girls are highly dependent on the decisions of men for their everyday activities, and their mobility is also limited unless a male accompanies them.^9^ The Baloch tribal system has a marriage system based on polygyny and exchange of women for marriages. Feudal anarchies among clans and sub-clans limit mobility of men, leading to increased immobility and suffering of women.^10^

There is a desperate need to identify and remedy the factors related to the poor performance of the health system and to learn about community behaviours related to low utilization of ANC that can support better planning and implementation of policies in future. The objective of the study was to determine factors associated with utilization of ANC services in Balochistan Province by analysing MICS 2010 data.

## METHODS

### Data

Data on utilization of ANC, together with social and economic determinants, were derived from a MICS conducted in Baluchistan, Pakistan in 2010 to assess important indicators related to community development and health in the province’surban and rural areas over its six regions on an overall basis. The survey used two-stage stratified sample design. In the first stage of sampling, 844 primary sampling units (PSU) (241 urban and 603 rural) were selected. The clusters in each district of a region were selected with probability proportional to their size. In the second stage, 12 households from each urban PSU and 16 households from each rural PSU were selected using systematic random sampling procedure. The questionnaires were based on the MICS-4 model questionnaires and were modified according to the Balochistan social and cultural norms and aligned with prevalence of certain diseases like HIV/AIDS. Only Pakistani citizens were included in the survey; 18,958 women 15-49 years old were identified as eligible for interview, and 17,732 were successfully interviewed yielding a response rate of 94%. Notably the data was adjusted for multistage sampling designs. No ethical approval was needed as this analysis was based on secondary data of MICS Balochistan 2010. However the Planning and Development Department, Government of Balochistan, granted permission for secondary analysis and the analysis was conducted from December 2014 to February 2015.

In this analysis the outcome variable were at least one ANC visit by a skilled provider in the last pregnancy and frequency of ANC visits in the two years preceding the survey. The initial analysisincluded indicators from household questionnaire and indicators from the questionnaire of individual women making any possible relation with ANC. Some of the variables were computed for binomial response converting multiple variables into one variable. In the survey spouse violence was asked in five domains but in this analysis the variable was computed in one variable as any violence by spouse.

The skilled provider in this analysis was a qualified health person like a doctor, Leady health worker (LHW), staff nurse and community midwife (CMW) or lady health visitor (LHV). Age of women at first marriage refers to the age that women got married for the first time. Number of children means the total number of children at the time of interview. Literate vs. Illiterate determines women having any formal education who at least can read and write a sentence. Higher wealth index score is the higher index score for the households calculated by MICS based on household assets. Symptoms of new-born at birth that needed hospitalization: refer to health status of new-borns who are born at home and spouse violence refers to husbands beating the women due to domestic/social problems.

### Statistical data analysis

The researchers have constructed two models to describe the behaviour of utilization of ANC. In the first model, researchers considered the women having ANC as a primary response (Y_1_). This was assumed to be associated with15 different factors (X) derived from the MICS, representing different socio-economic living conditions and health services utilization. The response (Y_1_) and factors matrix (X) are supposed to be linked in non-linear relation. The response is two class categorical variable presenting the utilization of ANC (yes or no). It is more appropriate to use logistic regression for the classification, where response is assumed to follow the binomial distribution. In logistic regression the probability of a women to have ANC i.e. p= P_r_ (y_1_ = 1) can be modelled as p = e^α^+^Xβ^ / 1-e^α+Xβ^

The parameter vector β = (α,β_1_ ,...,β_p_)’ is usually estimated by maximizing the log likelihood L(y_1_; β)=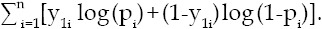


In the second model, the frequency of a woman having ANC utilization was considered as the response (Y_2_), which was assumed to be associated with socio-economic factors (X). The response here is the frequency of women availing themselves of ANC, which is normally assumed to follow the Poisson distribution or negative binomial distribution. Since the Poisson distribution assumes the homogeneity of response for the classes, while the negative binomial I sa more general form of Poisson distribution and does not require the homogeneity of the response for classes. Hence, we assume the frequency of ANC utilization follows the negative binomial distribution.

For both models, the researchers were mostly interested in constructing a parsimonious classification model, where noise and insignificant factors (X-variables) are removed from the model with targets to improve model classification performance and to increase the model interpretation. Notably, model classification performance is simply the percentage of correctly classified samples on test data. The researchers used the Akaike information criterion (AIC) and Bayesian information criterion (BIC) for the parsimonious model selection.^11^,^12^

### Statistical Software

All computations and model fitting was conducted through freely available statistical software ‘R’(www.r-project.org/).

## RESULTS

The researchers selected the 15 factors form MICS-2010 to use in Baluchistan, Pakistan, based on a literature review and available experience concerning factors that possibly influence pregnant women in deciding to obtain ANC services. The analysis was conducted by using 2339 samples where 47% of the women went for ANC. Both AIC and BIC-based selection was utilized. The AIC-based model identifies six influential factors with AIC=2883 and the BIC-based model identifies five influential factors with BIC=2888. Furthermore, both models were compared through ANOVA. The AIC-based selected model is presented in Table-I, where selected factors, their estimates, exponents of the estimates ‘exp(estimate)’ and standard error are listed. Factor’s estimates of logistic regression are hard to interpret directly, and a convenient way is to use exponent of estimates, which defines the effect of respective factors on the ‘odd ratio’ (the ratio between the probability of class 1 and 2).

**Table-I:** Final model (based on AIC) of factors associated with ANC utilization.

Factors	Estimate	exp (Estimate)	Std. Error
Intercept	-0.411	0.65	0.306
Literacy	1.049***	2.85	0.166
Number of children	-0.074*	0.92	0.033
Younger age of women at first marriage	-0.015**	0.98	0.005
Symptom of newborn at birth that needs hospitalization	-0.453*	0.64	0.248
Wealth index score	0.547***	1.72	0.051
Spouse violence	-0.359**	0.69	0.113

p-value =

*** 0.01,

** 0.05,

* 0.1

Within the group, literate pregnant women were 2.85 times more likely to access ANC than illiterate pregnant women. Pregnant women from rich families are 1.72 times more likely to go for ANC. Women with an increase in the number of children are 0.92 times more likely not having ANC. Women giving birth to new-borns developing symptoms needing hospitalization decreases their tendency in the future to get ANC by 0.64 times, and those women who think their spouse violence is socially justified are 0.69 times more likely to avoid ANC. Women getting married at younger ages are 0.98 times less likely to go for ANC.

Furthermore, to capture possible factors that may increase the frequency of a woman’s ANC visits, 863 samples of those who went for ANC were chosen, having a mean visit frequency of 3.01 and a median visit frequency of 3. The researchers used generalized linear model (GLM) for modelling the frequency of women’s visits for ANC, where response followed the negative binomial distribution. For final selection two criterions, including AIC and BIC were used. The researchers ran both AIC and BIC-based model selection. The AIC-based model identified 5 influential factors with AIC=3312, and the BIC-based model selection identified 3 influential factors with BIC=3317. Furthermore, both models were compared through ANOVA, and it was found that the AIC-based model out-performed BIC-based model significantly (p< 0.01). The AIC-based model is presented in Table-II, where selected factors, their estimates, exponent of the estimates ‘exp(estimate)’ and standard error are listed. The exponated frequency of ANC visits coefficient is the multiplicative term to calculate the estimated respective factor when frequency of ANC visits increases by 1 unit.

**Table-II:** Final model (AIC based) factors associated with frequency of ANC visits.

Factors	Estimate	exp (Estimate)	Std. Error
Intercept	0.887***	2.43	0.049
Source of ANC			
ANC from Doctor	-0.103*	0.91	0.057
Assistance at Birth			
Assistance at delivery by Doctor	-0.184***	0.83	0.046
Assistance at delivery by Nurse	0.210***	1.24	0.065
Components of ANC			
Urine sample taken at ANC	0.097***	1.11	0.023
TT injection before last pregnancy	0.137**	1.15	0.061

p-value =

*** 0.01,

** 0.05,

* 0.1

To assess the factors influencing frequency of ANC visits, Obtaining ANC was considered from a doctor, staff nurse, and LHW or other health personal was considered. However in the final model only obtaining ANC from a doctor showed significant association. However having ANC from a doctor showed decrease in frequency of women’s overall ANC visits by 0.91 times. Obtaining Doctor assistance at birth in last pregnancy also decreases the frequency by 0.83 times. Obtaining assistance by nurse at birth increases frequency of ANC visits by1.15 times. Women who went for urine sample increased their frequency of obtaining ANC by 1.11 times. Use of medication like tetanus toxoid (TT) injection during the last pregnancy increased the frequency of ANC visits by 1.15.

## DISCUSSION

This study revealed low prevalence of obtaining any ANC from a public or private health facility, a doctor, a TBA or any other health staff. The importance of the analysis can be assumed beneficial for the planning to improve maternal health as no study has been conducted before to assess the factors influencing ANC utilization and MICS Balochistan 2010, is the is the only survey conducted throughout province.

The study showed negative association between ANC and marriage at younger age. Previous studies have shown mixed results when relating age at first marriage and obtaining ANC in the rural areas of developing regions.^13^ There is evidence that women in their late reproductive life are more likely to have ANC than teenagers^14^ this is may be due to more number of children and perception of having more knowledge about complications related to pregnancy and during birth.

The researchers found that an increase in the number of children was associated with decreased ANC. Other studies elsewhere have shown less ANC among women who had an increased number of children.^15^ Frequent inequalities in maternal and child health care persist in the developing world. Education and household wealth are significantly associated with ANC utilization.^16^ It is evident that female literacy can increase the ‘need’ side factors to obtain health care but doubtlessly cannot balance shortages in supply of services. These shortages can be remedied through higher levels of government expenditure and participation in healthcare that will promote higher utilization rates of maternal health services.^17^

This study demonstrated that wealthier pregnant women obtained more ANC than pregnant woman in lower wealth families. Different studies confirm that people from lower income strata have poor understanding and utilization of preventive, promotive and curative aspects of health care services. They are negatively associated with positive health outcomes and good health status.^18^,^19^ Women in Balochistan who took their neonates to a health facility during last pregnancy because they developed any illness symptom at birth were obtaining less ANC services. A study in Balochistan found negative perceptions resulting from visiting low-standard health facilities in previous pregnancies.^20^ This indicates that healthcare providers are unable to provide required primary health care services in the community resulting in underutilization of the services provided by the government.^20^ Such a situation requires improving health services delivery in proper ways so pregnant mothers can be attracted to ANC services.

In the present analysis women who justified spouse violence socially (for reasons such as going out without telling her husband, neglecting children, arguing or burning the food) have low utilization of ANC services. In general, pregnant women who experience spouse violence have poorer health and complicated pregnancies and have much less access to health facilities, and such women use a disproportionate amount of health care services, including primary health care.^21^

Studies have shown that ANC from doctors and traditional birth attendants (TBA) are the lead ANC providers.^22^ However, this analysis has revealed that pregnant women who went for ANC and got assistance for birth from a doctor had fewer ANC visits. The reason behind this finding may be the increased cost of visiting the doctor and difficulties in accessibility.^20^ In Pakistan primary health care services are provided through other health personnel including community health workers such as lady health visitors (LHV), community midwifes (CMW) and lady health workers (LHW). The involvement of these other health personnel did not show any significant associations with the number of ANC visits. It is clear that the quality of services provided by doctors and nurses can increase number of visits during ANC 22,23 Components of ANC like urine sampling and provision of a TT injection at the health facility resulted in increased frequency of visits among pregnant women. The relationship between TT injection and increased number of visits may be the result of strong beliefs of the Pakistani population in the fast action of injections.^24^

## CONCLUSION

The results of analysis showed that ANC participation rate was low in the province. Pregnant women who had high wealth index scores had more chances to obtain ANC. Factors such as age of the women at marriage, number of living children, symptoms of any illness to neonates at birth that needed hospitalization and women who justify any spouse violence indicate significantly low ANC utilization. Components of ANC such as urine sample and TT injection given during last pregnancy increased the frequency of visits. ANC and assistance for birth from a doctor decreased the number of visits. Findings of this study suggest that a multidimensional approach is needed to decrease gaps among poor and rich and educated and illiterate in the underdeveloped and rural communities. The approach should be organized to identify problems and goals, mobilize resources and develop and implement strategies to achieve good health for pregnant mothers. A holistic approach in the future can deal successfully with social determinates of health and ensure healthy pregnancies among women living in rural areas like Balochistan which persistently have low ANC participation rates.
